# Successful Dissemination of Plasmid-Mediated Extended-Spectrum β-Lactamases in Enterobacterales over Humans to Wild Fauna

**DOI:** 10.3390/microorganisms9071471

**Published:** 2021-07-09

**Authors:** Racha Beyrouthy, Carolina Sabença, Frédéric Robin, Patricia Poeta, Giberto Igrejas, Richard Bonnet

**Affiliations:** 1 Institut National de la Santé et de la Recherche Médicale, (UMR1071), Institut National de la Recherche Agronomique (USC-2018), Université Clermont Auvergne, 63000 Clermont-Ferrand, France; rbeyrouthy@chu-clermontferrand.fr (R.B.); frobin@chu-clermontferrand.fr (F.R.); 2 Centre National de Référence de la Résistance aux Antibiotiques, Centre Hospitalier Universitaire, 63000 Clermont-Ferrand, France; 3 MicroART-Antibiotic Resistance Team, Department of Veterinary Sciences, University of Trá-os-Montes and Alto Douro (UTAD), 5001-801 Vila Real, Portugal; carolinasabenca@hotmail.com (C.S.); ppoeta@utad.pt (P.P.); 4 Department of Genetics and Biotechnology, University of Trás-os-Montes and Alto Douro, 5001-801 Vila Real, Portugal; gigrejas@utad.pt; 5 Functional Genomics and Proteomics Unit, University of Trás-os-Montes and Alto Douro, 5001-801 Vila Real, Portugal; 6 Associated Laboratory for Green Chemistry (LAQV-REQUIMTE), University NOVA of Lisbon, 2825-168 Caparica, Portugal

**Keywords:** *Escherichia coli*, β-lactamase, plasmid, CTX-M-1, IncI1-ST3

## Abstract

Background: The emergence of multidrug-resistant bacteria remains poorly understood in the wild ecosystem and at the interface of habitats. Here, we explored the spread of *Escherichia coli* containing IncI1-ST3 plasmid encoding resistance gene *cefotaximase-Munich-1* (*bla*_CTX-M-1_) in human-influenced habitats and wild fauna using a genomic approach. Methods. Multilocus sequence typing (MLST), single-nucleotide polymorphism comparison, synteny-based analysis and data mining approaches were used to analyse a dataset of genomes and circularised plasmids. Results. CTX-M-1 *E. coli* sequence types (STs) were preferentially associated with ecosystems. Few STs were shared by distinct habitats. IncI1-ST3-*bla*_CTX-M-1_ plasmids are disseminated among all *E. coli* phylogroups. The main divergences in plasmids were located in a shuffling zone including *bla*_CTX-M-1_ inserted in a conserved site. This insertion hot spot exhibited diverse positions and orientations in a zone-modulating conjugation, and the resulting synteny was associated with geographic and biological sources. Conclusions. The ecological success of IncI1-ST3-*bla*_CTX-M-1_ appears less linked to the spread of their bacterial recipients than to their ability to transfer in a broad spectrum of bacterial lineages. This feature is associated with the diversity of their shuffling conjugation region that contain *bla*_CTX-M-1_. These might be involved in the resistance to antimicrobials, but also in their spread.

## 1. Introduction

In recent decades, the consumption of antimicrobials has been rising in both humans and animals, and as a result, so has the prevalence of plasmid-mediated extended-spectrum β-lactamases (ESBLs) [1]. However, ESBLs confer resistance to penicillins and cephalosporins, including last-generation cephalosporins, which are key molecules for treating infections caused by Gram-negative bacteria in hospitals [1]. Consequently, the last-generation cephalosporins are classified by the World Health Organization (WHO) as critically important antimicrobial agents in human medicine [2]. The ESBLs are inhibited by clavulanic acid, sulbactam and tazobactam, and they are not efficient against carbapenem antimicrobials. Their main reservoir is Enterobacterales, especially the widespread and versatile species *Escherichia coli*, which is one of the intestinal microbiota and a major pathogen in humans and animals.

Antimicrobial resistance (AMR) is a complex and multifaceted threat to humans, animals, and the environment. A major cause of the AMR burden is the capability of resistant bacteria such as *E. coli* and AMR-encoding genes to spread between individuals, including across sectors by horizontal gene transfer. Plasmids are extra-chromosomal mobile genetic elements that play an essential role in bacterial ecology and evolution and they help their hosts adapt to a multitude of environments [3]. Plasmids carry accessory genes, including most clinically relevant resistance genes, such as those encoding carbapenemases [4], cephalosporinases [5] and the widespread ESBLs [6,7,8] that can spread across high-risk bacterial clones [9,10]. The most frequently detected ESBLs are class A β-lactamases. They represented by three major types: cefotaximase-Munich (CTX-M), temoneira (TEM) and sulfhydryl variable (SHV) and they include more than 400 variants reported today. These corresponding genes are often associated with other genes that confer resistance to beta-lactams and other antimicrobial agents such as quinolones, aminoglycosides and sulfonamides [7,8]. 

Initially, ESBLs were variants of TEM- and SHV-type penicillinases that acquired hydrolytic activity against oxyimino cephalosporins, also called third- and fourth-generation cephalosporins (C3G/C4G) through 1- to 4-point mutations. These enzymes were mainly observed during the 1980s and the 1990s in nosocomial Enterobacterales, such as *Klebsiella pneumoniae* and *Enterobacter cloacae*, which are mainly responsible for infections in immunocompromised patients in intensive care units [6,7,8]. Since the early 2000s, CTX-M-type ESBLs have been the dominant ESBLs all over the world, owing to their strong association with the species *E. coli*. This recipient, which is a major pathobiont of the mammal gut, favours the spread not only in intensive care units, as observed for TEM- and SHV-type ESBLs, but also in all other care units of hospitals and the community [5,6,7]. Consequently, the CTX-M-type ESBLs, especially variants CTX-M-15 and CTX-M-1, are community-acquired ESBLs, which have almost substituted for the TEM- and SHV-type ESBLs, and they are the most common plasmid-mediated ESBL among Enterobacterales isolates of human and veterinary origin worldwide [11,12,13,14,15]. CTX-M-15 is encoded by genes located in IncF plasmids harboured by *E. coli* ST131 clade C, a clade strongly associated with human hosts. CTX-M-1 is observed in *E. coli* strains collected from humans and animals, and its gene *bla*_CTX-M-1_ has been associated mainly with the broad host range IncN plasmids, and much more frequently in the narrow host range IncI1 [16,17,18,19,20,21,22,23,24,25]. 

The IncI plasmids belong to the I-complex plasmid family including the incompatibility groups IncI1, IncIγ, IncB, IncZ and IncK [26]. The IncI1 plasmid backbone is organised into four major conserved regions encoding replication, conjugative transfer, stability and leading [27,28], in addition to variable regions encoding accessory functions such as antimicrobial gene resistance.

There is a great concern that contacts with animals may enhance the risk of acquiring ESBL-encoding plasmids by humans [29,30]. IncI1-ST3 plasmids are one of the most prevalent plasmids in ESBL CTX-M-1 in Enterobacterales isolated from humans, animals and environmental sources [18,19,20,21,22,23,24,25]. However, the relationships at the interface of humans and animals remain elusive, especially for wild animals. This study compared CTX-M-1-producing *E. coli* isolates and IncI1-ST3 plasmids collected from humans, food-producing animals, and wild animals to best understand the CTX-M-1 spread among these ecosystems.

## 2. Materials and Methods

Genomic dataset. For this study, we collected 122 *E. coli* whole genome sequences (WGSs) containing IncI1-ST3 plasmids and *bla*_CTX-M-1_ (Supplementary Tables S1 and S2). The dataset includes WGSs sequenced during this study (*n* = 43) and recovered from GenBank (*n* = 79) after filtering for quality (ATCG assembly size >4.5 Mb, contigs number <200 and N50 > 60,000) and the availability of metadata. The sources of strains were humans (*n* = 57), domestic animals (*n* = 11), food or food-producing animals (*n* = 37), wild animals (*n* = 13) [20,31,32,33,34,35,36,37] and municipal wastewater (*n* = 1). Three strains were from unknown origins. Of this collection, 30 human strains and 13 strains isolated from wild animals were sequenced for this study (Supplementary Table S1). The other data were collected from the NCBI Short Read Archive (SRA) or the European Nucleotide Archive (ENA) by screening of the *E. coli* genomes of the GenBank database (Supplementary Table S2). The screening for encoding CTX-M-1- and IncI1-ST3-specific alleles was performed with DIAMOND and blastn software, respectively, using 100% identity threshold and 100% coverage threshold. 

Likewise, 20,668 non-redundant complete plasmids collected from GenBank were screened for the IncI1-ST3 feature and the presence of *bla*_CTX-M-1_. It resulted in a collection of 39 IncI1-ST3-*bla*_CTX-M-1_ circularised plasmids (Supplementary Table S3).

Whole genome sequencing (WGS). This was performed using the next-generation sequencing platform of the teaching hospital of Clermont-Ferrand, France. DNA was extracted with a DNeasy UltraClean Microbial kit (Qiagen, Hilden, Germany). The libraries were prepared with a Nextera XT Kit (Illumina, San Diego, CA, USA), and they were sequenced by the Illumina MiSeq system, generating 2 × 301-base pair (bp) paired-end reads. Fastp software v0.19.10 [38] was used for quality filtering of Illumina reads, and SPAdes was used for short reads assembly [39]. The mean sequencing depth was ≥163×; the number of assembled contigs ranged between 51 and 175, the mean contig number was 99.77, the N50 ranged between 63,075 and 383,707, and the mean contig number was 186,502. The genome sizes ranged between 4,667,864 and 5,338,201 nucleotides. The raw reads have been deposited in the European Nucleotide Archive (ENA, https://www.ebi.ac.uk/ena) under project accession number PRJEB36175.

Molecular typing. *E. coli* phylogroups and multilocus sequence typing (MLST) were determined in silico according to the Clermont Typing method [40] and Achtman’s MLST scheme [41]. The molecular typing of isolates was performed by core genome SNP-based typing (cgSNP). BactSNP was used to perform cgSNP using the *E. coli* core genome downloaded from the Enterobase website (https://enterobase.warwick.ac.uk) as a reference, as previously described [42,43]. After the filtration of recombination zones detected by Gubbins [44], a phylogenetic tree was inferred from the resulting alignment by maximum likelihood using RAxML [45]. 

Antimicrobial gene detection. The antimicrobial-resistant genes were identified by alignment against a database including the online databases CARD [46], Resfinder [47], and the NCBI National Database of Antibiotic Resistant Organisms (https://www.ncbi.nlm.nih.gov/pathogens/antimicrobial-resistance/ (accessed on 1 April 2021)) using a 95% minimum threshold for the breadth of coverage and identity percentage, as previously described [48].

Synteny analysis. This was performed with the Sibelia package [49]. The presence/absence matrix inferring from the resulting synteny blocks was analysed by multiple correspondence analysis (MCA) and hierarchical clustering (HC) in R with package FactoMiner (https://www.r-project.org).

## 3. Results

### 3.1. Whole Genome Typing of *E. coli* Harbouring Plasmids IncI1-ST3 and bla_CTX-M-1_

**Figure 1:**
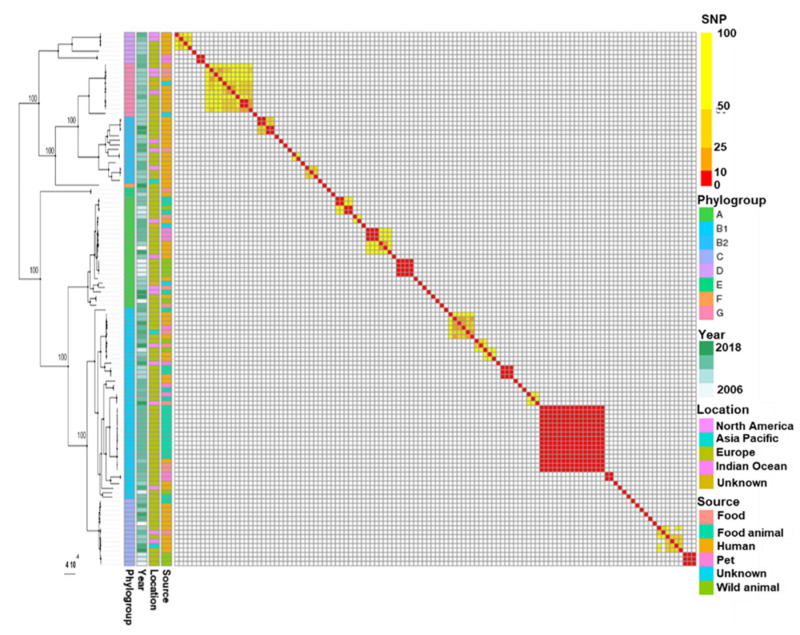
Phylogenetic tree and corresponding distance matrix based on SNPs of *E. coli* WGSs containing plasmids IncI1-ST3 encoding *bla*_CTX-M-1_. SNP calling, SNP filtering and tree inferring were performed with bactSNP, Gubbins and RAxML, respectively. The bootstrap values are indicated for the major phylogenetic branches as percentages for 500 replications.

To best understand the large diffusion of the CTX-M-1 ESBL, we collected a dataset comprising 122 *E. coli* WGSs containing IncI1-ST3 replicon and *bla*_CTX-M-1_ isolated from humans, human-influenced habitats and wild fauna. The corresponding genomes were classified into eight major phylogenetic branches by SNP-based core genome typing. These major branches corresponded to the *E. coli* phylogroups (Figure 1).

Most genomes belonged to phylogroups B1 (35.8%, *n* = 43), A (20.8%, *n* = 25), B2 (12.5%, *n* = 15), C (12.5%, *n* = 15) and G (10%, *n* = 12). The distribution of genomes among the phylogroups significantly differed depending on the originating source (Fisher test, *p*-value < 0.001). Since the genomes of phylogroups A and B1 frequently originated from food or food-producing animals (50% and 67%, respectively), the B2 genomes preferentially originated from humans (93.3% versus 6.7% from food and food-producing animals).

**Figure 2:**
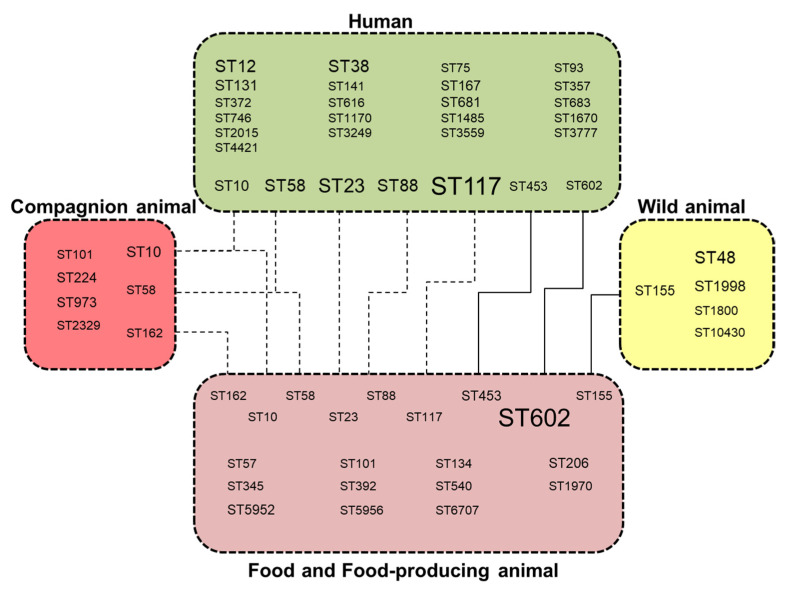
Distribution of *E. coli* ST lineages in human, human-influenced habitats and wild fauna showing deduced transmission pathways between these ecosystems. Dashed lines indicate STs shared by different habitats, and solid lines indicate closely related isolates diverging by ≤10 SNPs.

Human *E. coli* strains (*n* = 51/57) were distantly related, except for three clusters of two strains diverging by ≤10 SNPs and belonging to ST117 (*n* = 2) and ST12 (*n* = 2 × 2). The clonal isolates (divergence ≤ 10 SNPs) mainly clustered isolates from food and animals including wild animals. Few clonal clusters and STs contained isolates from different habitats (Figure 2), with possible cross-transmissions between humans and human-influenced habitats, and between wild and food-producing animals.

### 3.2. Antimicrobial Resistance Genes

**Figure 3:**
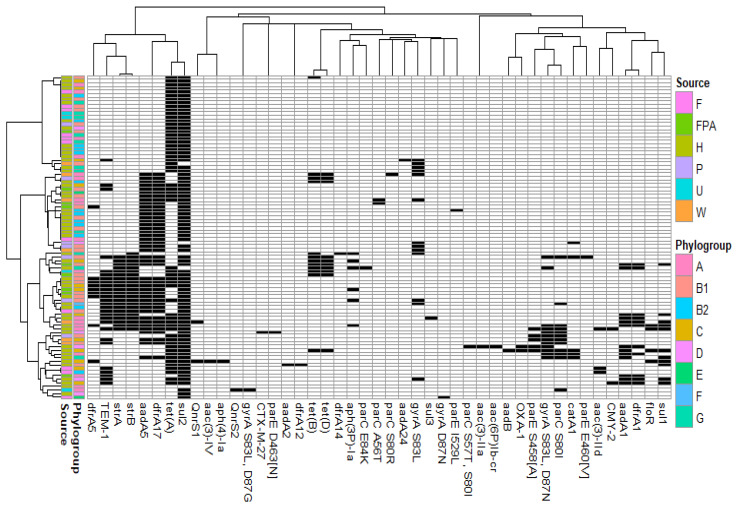
Antimicrobial resistance mechanisms associated with non-redundant *E. coli* harbouring *bla*_CTX-M-1_ and IncI1-ST3 plasmids (black: presence; white: absence). Source: F, food; FPA, food-producing animals; H, human; P, pet; U: unknown and W, wild animal.

In addition to the chromosome-mediated ampC gene encoding cephalosporinase, 45 acquired antimicrobial resistance mechanisms were associated with *bla*_CTX-M-1_ (Figure 3). None of the genes were strictly conserved. 

Excluding the redundant isolates corresponding to clonal isolates (*n* = 30), the most frequent genes were sulphonamide resistance gene *sul2* (94.4%), tetracycline resistance gene *tet(A)* (63.3%), streptomycin/spectinomycin resistance genes *aadA5* (47.8%), *strB* (24.4%) and *strA* (23.3%), trimethoprim resistance gene *dfrA17* (47.8%), and penicillinase-encoding gene *bla*_TEM-1_ (30.0%) (Supplementary Figure S1a). The investigation of resistance gene co-occurrence revealed preferential associations. Among the most frequent genes, *aadA5*, *dfrA17*, *strA*, *strB* and *bla*_TEM-1_ exhibited a strong association index. This suggests their frequent coexistence with *bla*_CTX-M-1_ probably in the same plasmid IncI1-ST3 (Supplementary Figure S1b).

### 3.3. SNP Analysis of bla_CTX-M-1_-Encoding Plasmids IncI1-ST3

**Figure 4:**
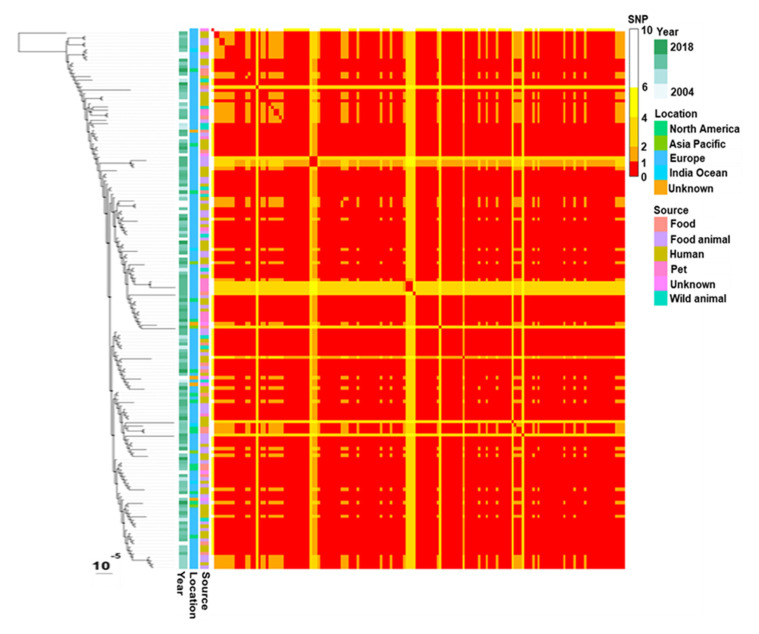
Phylogenetic tree and corresponding distance matrix based on SNPs of plasmids IncI1-ST3 encoding *bla*_CTX-M-1_. SNP calling, SNP filtering and tree inferring were performed with bactSNP, Gubbins and RAxML, respectively.

A total of 117 assemblies (96%) contained a contig harbouring *bla*_CTX-M-1_, IS*Ecp1* and at least the B segment of a region previously called shufflon that is specific to IncI1 plasmids [50]. The well-known mobile block IS*Ecp1*-*bla*_CTX-M-1_ [51,52] was located 333 pb upstream of the B segment of the shufflon. In four cases, mobile element IS*Kpn26* was inserted between *bla*_CTX-M-1_ and IS*Ecp1*. The *bla*_CTX-M-1_ gene was encoded by plasmids IncI1 in most *E. coli* harbouring this family of plasmids. SNP analysis of IncI1-ST3 plasmids encoding *bla*_CTX-M-1_ showed that they differ by <10 SNPs and most often by 1–2 SNPs. The resulting tree had a comb-like shape constituting a unique major clade (Figure 4).

### 3.4. Synteny Variation in bla_CTX-M-1_-Encoding Plasmids IncI1-ST3

**Figure 5:**
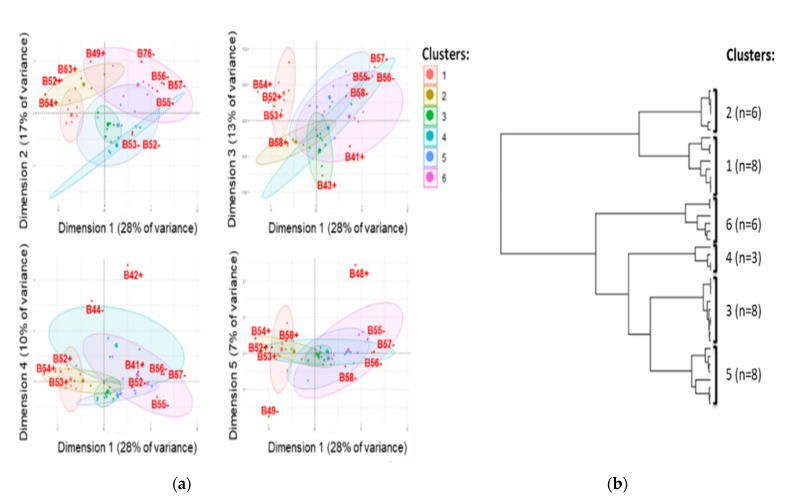
Multiple corresponding analysis (**a**) and hierarchical clustering (**b**) inferring synteny analysis of 39 circularised plasmids IncI1-ST3 encoding *bla*_CTX-M-1_. The clusters are surrounded by 95% confidence ellipses, and the 10 most contributory synteny blocks of sequences are indicated in red (B#: shuffling segment B associated with IS*Ecp1* and *bla*_CTX-M-1_; #: position of the genetic feature, rv: reverse and fd: forward).

Although not explored for epidemiologic investigations, genetic rearrangements are a major driving force of plasmid evolution. Therefore, we investigated synteny variations in 39 circularised IncI1-ST3-*bla*_CTX-M-1_ plasmids. The synteny analysis by multiple correspondence analysis (MCA) and hierarchical clustering (HC) classified the plasmids into six major clusters (Figure 5). The clusters are supported by statistical tests (Adonis test’s *p*-value: 0.001 and R^2^: 0.75; Dispersion permutation test’s *p*-value: 0.1). 

Among the 14 synteny blocks that were significantly associated with the clusters (FDR-adjusted X2-test’s *p*-values, 1.4 × 10^−6^ to 5.5 × 10^−3^), 13 were in the unique shufflon region between the conserved genes *rci* and *pilV*. The genetic features in region *rci*-*pilV* are specific to IncI1 plasmids, and they comprise up to four DNA segments A to D, previously identified as randomly rearranged by recombinase Rci [50]. This region harboured the most synteny variations. 

The synteny of *rci*-*pilV* was also investigated from *E. coli* WGSs. A total of 77 WGS-encoding plasmids IncI1-ST3 exhibited a complete shufflon assembly, including *bla*_CTX-M-1_. MCA and HC analyses of synteny variants from WGSs confirmed the classification of plasmids in six major clusters (Supplementary Figures S2–S4). Segment D was absent in all plasmids, and mobile element IS*Ecp1*-*bla*_CTX-M-1_ was always located downstream from segment B to form a conserved block exhibiting different positions in the shufflon. This block can affect the shuffling process, and it was paradoxically the feature that contributed most to diversity (Supplementary Figure S2). The shufflon segments are involved in the synthesis of PilV adhesins of the conjugative pilus [53]. Therefore, the shuffling of segments associated with IS*Ecp1*-*bla*_CTX-M-1_ insertion generates diversity in the PilV-encoding region. This can modulate the recognition of recipient cells during IncI1-ST3-*bla*_CTX-M-1_ conjugation and therefore probably their dissemination.

At the highest level of classification resolution, synteny analysis revealed 16 clusters of two to seven plasmids sharing identical synteny (Supplementary Figures S3 and S4). Ten of these clusters included plasmids isolated from the same country and the same source. Seven clusters were specific to the source. Nine clusters were only observed in human-influenced habitats. The plasmids isolated from wild animals were included in four clusters; two were specific to wild fauna, and the two others supported a possible spread between human-influenced animals and wild fauna. 

## 4. Discussion

The increase in antimicrobial resistance worldwide is a result of inappropriate use of antimicrobials during the last decades, including those used for human medication and animal husbandry. This broad use increases the selective pressure on both commensal and pathogenic bacteria, which can spread between different ecosystems [1,54]. Livestock animals may act as reservoirs of AMR and multidrug resistant bacteria. This can lead to dissemination of AMR into humans directly by contact and the food chain or indirectly from the environment [1,54].

In this study, we analysed genomic data belonging to *E. coli* isolates collected from humans, animals (food-producing animals, companion animals, wildlife), and food samples to understand the interaction between these ecosystems in the diffusion of IncI1-ST3 plasmids encoding *bla*_CTX-M-1_. The genomic data analysis showed that the *E. coli* phylogroups harbouring IncI1-ST3 plasmids encoding *bla*_CTX-M-1_ significantly differed depending on the originating source. Of the isolates, 68% belonged to the phylogenetic A, B1 and C, which are associated with multiple antimicrobial resistance genes especially those encoding sulphonamide and tetracycline resistance. MLST revealed 50 sequence types. The most abundant sequence type was ST602, followed by ST117 and ST10. The correlation between ST602, which was the most abundant sequence type detected in *E. coli* phylogenetic group B1 isolates in this study, and food-producing animals was pointed out in recent reports [55,56,57]. 

Human contamination by ESBL-producing Enterobacterales from animals is often supposed, and food is considered a direct transmission vehicle. ESBL gene *bla*_CTX-M-1_ and IncI1-ST3 plasmids were widespread in humans, human-influenced habitats and wild fauna, as previously observed [21,22,23,24,25]. Here, we observed that IncI1-ST3-*bla*_CTX-M-1_ plasmids also have disseminated into all *E. coli* lineages, which cover a broad diversity of bacteria and different lifestyles, including commensal and pathogenic strains. Few clonal clusters and STs were shared by different habitats, suggesting *E. coli* lineages have a preferential habitat and few of them are involved in cross-sector spread. As shuttles, these subgroups may be risk factors for spreading antimicrobial resistance and they might be preferential targets for strategies to prevent the spread of antimicrobial resistance.

The SNP-based comparison of *bla*_CTX-M-1_ IncI1-ST3 plasmids originating from several continents revealed a core genome highly conserved. This suggests that the dissemination of these plasmids across all sources over distant areas took decades. However, the evolutionary rate of bacterial genomes may not generate enough variations to resolve recent epidemiological processes involving small genetic elements such as IncI1-ST3-*bla*_CTX-M-1_ plasmids. Recombination, gain and loss of DNA fragments are key processes of evolution [58]. They affect synteny and are not investigated by comparisons based on core genome SNPs. The analysis of synteny in complete *bla*_CTX-M-1_ IncI1-ST3 plasmids revealed more diversity than SNP analysis. Synteny-based clusters were associated with sampling sources and geographic origins. This suggests that synteny analysis can be a useful approach for monitoring IncI1-ST3 plasmid spread over short periods, and it might help to analyse transmission chains.

Most variations in synteny were observed in a single region, which was previously designated shufflon and encoding a recombinase and targeted DNA segments [50]. Most diversity resulted from the positioning and orientation of a conserved block including the shufflon segment B, IS*Ecp1* and *bla*_CTX-M-1_. Shufflon B appears, therefore, as a hot spot for the insertion of mobile element IS*Ecp1* and associated gene *bla*_CTX-M-1_. Since IS*Ecp1* is involved in the mobilisation of ESBL- and cephalosporinase-encoding genes [51,59], shufflon B of IncI1 explains the key role of these plasmids in the spread of resistance to last-generation cephalosporins. 

The assembly of *bla*_CTX-M-1_ from short reads suggest a certain stability and/or the preponderance of a shufflon synteny within a bacterial clone. This contrasts with plasmids IncI2 harbouring active shufflons [60]. This stability was confirmed by assembly from long-read sequencing, which did not reveal alternative conformation of shufflons [61] and may be explained by the insertion of IS*Ecp1*-*bla*_CTX-M-1_ in the shuffling zone. The shufflon is involved in the synthesis of PilV adhesins, which are responsible for recipient recognition in the conjugation process [53]. The insertion of IS*Ecp1-bla*_CTX-M-1_ associated with the shuffling of segments can affect *PilV* and consequently modulate the recognition of recipient cells during IncI1-ST3*-bla*_CTX-M-1_ conjugation. Resistance gene *bla*_CTX-M-1_ can, therefore, be involved in both antimicrobial resistance and plasmid spread, two synergic functions that may explain the ecological success of *bla*_CTX-M-1_ IncI1-ST3 plasmids.

## 5. Conclusions

Although additional animal and environmental sources of CTX-M-1-producing *E. coli* should be investigated, the results showed there was broad dissemination of IncI1-ST3-*bla*_CTX-M-1_ plasmids. Their bacterial recipients differ by habitats, with a few of them playing the role of disseminating shuttles. The sequence of IncI1-ST3-*bla*_CTX-M-1_ plasmids is highly conserved except in the shufflon zone. Their broad ecological success does not seem to be linked to their ability to transfer a broad spectrum of bacterial lineages, a feature associated with the diversity of their shuffling conjugation region.

## Data Availability

The accession numbers of genomic data are reported in Supplementary Tables S2 and S3.

## Supplementary Materials

The following are available online at https://www.mdpi.com/article/10.3390/microorganisms9071471/s1, Figure S1: Frequency (a) and correlation index (b) of antimicrobial resistance genes in non-redundant *E. coli* harbouring *bla*_CTX-M-1_ and IncI1-ST3 plasmids, Figure S2: Multiple corresponding analysis performed from the synteny of shuffling region of *bla*_CTX-M-1_ IncI1-ST3 plasmids, Figure S3: Heatmap showing the presence (red) and the absence (blue) of synteny blocks in the shuffling region of plasmids IncI1-ST3-*bla*_CTX-M-1_, Figure S4: Hierarchical classification of plasmids IncI1-ST3-*bla*_CTX-M-1_ based on the synteny of the shufflon region, Table S1: Metadata associated with the *Escherichia coli* isolates sequenced during this study, Table S2: Dataset of *Escherichia coli* whole genome sequences (WGSs) containing replicon IncI1-ST3 encoding *bla*_CTX-M-1_, Table S3: Circularised *bla*_CTX-M-1_-encoding plasmids IncI1-ST3 included in this study.
