# Supplementary material for: Successful Dissemination of Plasmid-Mediated Extended-Spectrum β-Lactamases in Enterobacterales over Humans to Wild Fauna
Source: Microorganisms. 2021 Jul 9;9(7):1471. doi: 10.3390/microorganisms9071471 (PMC8305760; doi:10.3390/microorganisms9071471)

Table S1: Metadata associated with the *Escherichia coli* isolates sequenced during this study.

Strain ID	Species	Sampling source	Sample	Country	Year
TB63	*E. coli*	Wild animal (Bat)	Stool	Portugal	2014
1-102	*E. coli*	Wild animal (Bird of prey)	Stool	Portugal	2008
13-103	*E. coli*	Wild animal (Bird of prey)	Stool	Portugal	2008
2-101	*E. coli*	Wild animal (Bird of prey)	Stool	Portugal	2008
21-101	*E. coli*	Wild animal (Bird of prey)	Stool	Portugal	2008
BU10A	*E. coli*	Wild animal (Buzzard)	Stool	Portugal	2007
BU10B	*E. coli*	Wild animal (Buzzard)	Stool	Portugal	2007
BU22A	*E. coli*	Wild animal (Buzzard)	Stool	Portugal	2007
BU41A	*E. coli*	Wild animal (Buzzard)	Stool	Portugal	2007
J31	*E. coli*	Wild animal (Wild board)	Stool	Portugal	2006
J64	*E. coli*	Wild animal (Wild board)	Stool	Portugal	2006
J69	*E. coli*	Wild animal (Wild board)	Stool	Portugal	2006
W4	*E. coli*	Wild animal (Wolf)	Stool	Portugal	2008
AURA86	*E. coli*	Human	Urine	France	2019
BLSE107-2012	*E. coli*	Human	Urine	France	2012
BLSE11-2012	*E. coli*	Human	Urine	France	2012
BLSE114-2012	*E. coli*	Human	Blood	France	2012

BLSE119-2018	*E. coli*	Human	Skin	France	2018
BLSE120-2012	*E. coli*	Human	Respiratory tract	France	2012
BLSE124-2012	*E. coli*	Human	Blood	France	2012
BLSE128-2012	*E. coli*	Human	Urine	France	2012
BLSE143-2012	*E. coli*	Human	Urine	France	2012
BLSE171-2012	*E. coli*	Human	Urine	France	2012
BLSE17-2012	*E. coli*	Human	Urine	France	2012
BLSE175-2012	*E. coli*	Human	Blood	France	2012
BLSE179-2018	*E. coli*	Human	Urine	France	2018
BLSE192-2012	*E. coli*	Human	Biopsie	France	2012
BLSE193-2018	*E. coli*	Human	Urine	France	2018
BLSE200-2012	*E. coli*	Human	Urine	France	2012
BLSE27-2012	*E. coli*	Human	Urine	France	2012
BLSE34-2012	*E. coli*	Human	Urine	France	2012
BLSE41-2018	*E. coli*	Human	Urine	France	2018
BLSE77-2012	*E. coli*	Human	Urine	France	2012
BLSE93-2012	*E. coli*	Human	Urine	France	2012
CNR1626	*E. coli*	Human	Stool	France	2016
CNR3515	*E. coli*	Human	Stool	France	2019
CVL138	*E. coli*	Human	Urine	France	2019

NC105	*E. coli*	Human	Blood	France (New Caledonia)	2014
PAP34	*E. coli*	Human	Urine	France (Guadeloupe)	2013
PAP37	*E. coli*	Human	Urine	France (Guadeloupe)	2013
PAP43	*E. coli*	Human	Respiratory tract	France (Guadeloupe)	2013
GHSR6	*E. coli*	Human	Urine	France (Reunion)	2013
PAP45	*E. coli*	Human	Urine	France (Guadeloupe)	2013

Table S2: Dataset of *Escherichia coli* whole genome sequences (WGSs) containing replicon IncI1-ST3 encoding *bla*CTX-M-1.

Biosample accession number	Achtman's Ecoli ST	Achtman's ST clonal complex	Phylogroup	Sampling source	Year	Country
SAMEA6471237 [a]	1998	ST23 Cplx	C	wild animal (wild boar)	2006	Portugal
SAMEA6471229 [a]	1998	ST23 Cplx	C	wild animal (Bird of prey)	2008	Portugal
SAMEA6471230 [a]	1998	ST23 Cplx	C	wild animal (Bird of prey)	2008	Portugal
SAMEA6471227 [a]	1998	NA [b]	C	Wild animal (Bird of prey)	2008	Portugal
SAMEA6471236 [a]	48	ST10 Cplx	A	wild animal (wild boar)	2006	Portugal
SAMEA6471231 [a]	48	ST10 Cplx	A	wild animal (Buzzard)	2007	Portugal
SAMEA6471232 [a]	48	ST10 Cplx	A	wild animal (Buzzard)	2007	Portugal
SAMEA6471233 [a]	48	ST10 Cplx	A	wild animal (Buzzard)	2007	Portugal
SAMEA6471238 [a]	155	ST155 Cplx	B1	wild animal (wolf)	2008	Portugal
SAMEA6471240 [a]	155	ST155 Cplx	B1	wild animal (bat)	2014	Portugal
SAMEA6471235 [a]	10430	ST168 Cplx	A	wild animal (wild boar)	2006	Portugal
SAMEA6471234 [a]	1800	NA [b]	B1	wild animal (Buzzard)	2007	Portugal
SAMEA6471228 [a]	1800	NA [b]	B1	Wild animal (Bird of prey)	2008	Portugal
SAMN02471512	453	ST86 Cplx	B1	Human	2011	Netherlands
SAMN09399352	3777	ST350 Cplx	E	Human	2011	France
SAMEA6823483 [a]	23	ST23 Cplx	C	Human	2012	France
SAMEA6823484 [a]	23	ST23 Cplx	C	Human	2012	France
SAMEA6823492 [a]	3249	ST23 Cplx	C	Human	2012	France
SAMEA6921714 [a]	10	ST10 Cplx	A	Human	2013	France (Guadeloupe)

SAMEA6823498 [a]	167	ST10 Cplx	A	Human	2012	France
SAMEA6823493 [a]	746	ST10 Cplx	A	Human	2012	France
SAMEA6823496 [a]	12	ST12 Cplx	B2	Human	2012	France
SAMEA6823497 [a]	12	ST12 Cplx	B2	Human	2012	France
SAMEA6823486 [a]	38	ST38 Cplx	D	Human	2012	France
SAMEA6823487 [a]	38	ST38 Cplx	D	Human	2012	France
SAMN04273082	131	ST131 Cplx	B2	Human	2012	United Kingdom
SAMN13023763	131	ST131 Cplx	B2	Human	2013	United Kingdom
SAMEA6823489 [a]	681	NA [b]	B2	Human	2012	France
SAMEA6823494 [a]	681	NA [b]	B2	Human	2012	France
SAMEA6823490 [a]	141	NA [b]	B2	Human	2012	France
SAMEA6823491 [a]	1670	NA [b]	D	Human	2012	France
SAMEA6921709 [a]	4421	NA [b]	B1	Human	2012	France
SAMEA6823499 [a]	117	NA [b]	G	Human	2012	France
SAMEA6823495 [a]	117	NA [b]	G	Human	2012	France
SAMEA6823488 [a]	117	NA [b]	G	Human	2012	France
SAMN05977355	117	NA [b]	G	Human	2013	Netherlands
SAMEA6921715 [a]	117	NA [b]	G	Human	2013	France
SAMN05977362	58	ST155 Cplx	B1	Human	2013	Netherlands
SAMN13023739	602	ST446 Cplx	B1	Human	2013	United Kingdom
SAMN05977341	58	ST155 Cplx	B1	Human	2012	Netherlands
SAMEA6921712 [a]	683	ST155 Cplx	B1	Human	2013	France (Reunion)

SAMEA6921716 [a]	23	ST23 Cplx	C	Human	2013	France (Guadeloupe)
SAMEA6921717 [a]	2015	NA [b]	B2	Human	2013	France (Guadeloupe)
SAMN14465408	38	ST38 Cplx	D	Human	2014	Canada
SAMEA6921713 [a]	1170	NA [b]	B2	Human	2014	France (New Caledonia)
SAMN13024512	117	NA [b]	G	Human	2014	United Kingdom
SAMN12145257	75	NA [b]	B1	Human	2015	France
SAMN12145271	616	ST155 Cplx	B1	Human	2015	France
SAMN07325904	131	ST131 Cplx	B2	Human	2016	USA
SAMN07325903	38	ST38 Cplx	D	Human	2016	USA
SAMN05774074	10	ST10 Cplx	A	Human	2016	USA
SAMN12145206	117	NA [b]	G	Human	2016	France
SAMN12145122	117	NA [b]	G	Human	2016	France
SAMN12145225	88	ST23 Cplx	C	Human	2016	France
SAMN12145164	357	NA [b]	B2	Human	2016	France
SAMN12145266	58	ST155 Cplx	B1	Human	2016	France
SAMN12145137	88	ST23 Cplx	C	Human	2017	France
SAMN12768772	23	ST23 Cplx	C	Human	2017	Australia
SAMN12145194	372	NA [b]	B2	Human	2017	France
SAMN09400798	3559	ST469 Cplx	B1	Human	2017	USA
SAMEA6921706 [a]	58	ST155 Cplx	B1	Human	2018	France
SAMEA6921707 [a]	10	ST10 Cplx	A	Human	2018	France

SAMEA6921708 [a]	88	ST23 Cplx	C	Human	2018	France
SAMEA6921705 [a]	12	ST12 Cplx	B2	Human	2018	France
SAMEA6921710 [a]	12	ST12 Cplx	B2	Human	2019	France
SAMEA6921711 [a]	1485	ST648 Cplx	F	Human	2019	France
SAMEA6921704 [a]	93	ST168 Cplx	A	Human	2019	France
SAMEA6378021	23	ST23 Cplx	C	Human	2019	France
SAMEA3512092	167	ST10 Cplx	A	Human	NS	NA [c]
SAMEA3673016	88	ST23 Cplx	C	Human	NS	NA [c]
SAMEA6471223 [a]	206	ST206 Cplx	A	Food producing animal (pig)	2008	Portugal
SAMN02471476	453	ST86 Cplx	B1	Food producing animal (pig)	2011	Netherlands
SAMN02471486	453	ST86 Cplx	B1	Food producing animal (pig)	2011	Netherlands
SAMN11958806	540	NA [b]	A	Food producing animal (pig)	2019	USA
SAMEA3753150	602	ST446 Cplx	B1	Food producing animal (chicken)	2015	United Kingdom
SAMEA3752738	602	ST446 Cplx	B1	Food producing animal (chicken)	2015	United Kingdom
SAMEA3753147	602	ST446 Cplx	B1	Food producing animal (chicken)	2015	United Kingdom
SAMEA3753148	602	ST446 Cplx	B1	Food producing animal (chicken)	2015	United Kingdom
SAMEA3753399	602	ST446 Cplx	B1	Food producing animal (chicken)	2015	United Kingdom
SAMEA3752316	602	ST446 Cplx	B1	Food producing animal (chicken)	2015	United Kingdom
SAMEA3752739	602	ST446 Cplx	B1	Food producing animal (chicken)	2015	United Kingdom
SAMEA3751405	602	ST446 Cplx	B1	Food producing animal (chicken)	2015	United Kingdom
SAMEA3752741	602	ST446 Cplx	B1	Food producing animal (chicken)	2015	United Kingdom
SAMEA3751406	602	ST446 Cplx	B1	Food producing animal (chicken)	2015	United Kingdom

SAMEA3752617	602	ST446 Cplx	B1	Food producing animal (chicken)	2015	United Kingdom
SAMEA3485084	602	ST446 Cplx	B1	Food producing animal (chicken)	2015	United Kingdom
SAMN12641083	392	NA [b]	B1	Food producing animal (bovin)	2016	France
SAMEA3993610	345	ST23 Cplx	B1	Food (pork)	2014	Denmark
SAMEA3993572	88	ST23 Cplx	C	Food (pork)	2014	Denmark
SAMEA3993566	101	ST101 Cplx	B1	Food (pork)	2014	Denmark
SAMN02463220	162	ST469 Cplx	B1	Food (pork)	2014	Denmark
SAMEA3753386	5952	ST206 Cplx	A	Food (pork)	2014	United Kingdom
SAMEA3752514	5952	ST206 Cplx	A	Food (pork)	2014	United Kingdom
SAMN13738180	58	ST155 Cplx	B1	Food (pork)	2017	South Korea
SAMEA6471221 [a]	206	ST206 Cplx	A	Food (chicken)	2008	Portugal
SAMN02471484	10	ST10 Cplx	A	Food (chicken)	2010	Netherlands
SAMN02390174	117	NA [b]	G	Food (chicken)	2012	USA
SAMN02390175	6707	NA [b]	A	Food (chicken)	2013	USA
SAMN07624461	23	ST23 Cplx	C	Food (chicken)	2015	USA
SAMN07624453	117	NA [b]	G	Food (chicken)	2013	USA
SAMEA3485102	5956	NA [b]	A	Food (chicken)	2015	United Kingdom
SAMEA3485100	602	ST446 Cplx	B1	Food (chicken)	2015	United Kingdom
SAMEA3485111	57	ST350 Cplx	E	Food (chicken)	2015	United Kingdom
SAMEA3485105	602	ST446 Cplx	B1	Food (chicken)	2015	United Kingdom
SAMN10221082	134	NA [b]	B2	Food (chicken)	2017	USA
SAMN12943438	155	ST155 Cplx	B1	Food (chicken)	2019	USA

SAMN12943740	101	ST101 Cplx	B1	Food (chicken)	2019	USA
SAMN12911910	224	NA [b]	B1	Domestic animal (cat)	2014	Poland
SAMN12911909	224	NA [b]	B1	Domestic animal (cat)	2014	Poland
SAMN08519283	58	ST155 Cplx	B1	Domestic animal (dog)	2015	Germany
SAMN08519253	973	NA [b]	D	Domestic animal (dog)	2015	Germany
SAMN08519245	973	NA [b]	D	Domestic animal (dog)	2015	Germany
SAMN08519214	101	ST101 Cplx	B1	Domestic animal (dog)	2015	Germany
SAMN08519216	10	ST10 Cplx	A	Domestic animal (dog)	2015	Germany
SAMN08519212	10	ST10 Cplx	A	Domestic animal (dog)	2015	Germany
SAMN08519209	10	ST10 Cplx	A	Domestic animal (dog)	2015	Germany
SAMN08519278	162	ST469 Cplx	B1	Domestic animal (dog)	2015	Germany
SAMN12601668	2329	NA [b]	B1	Domestic animal (dog)	2018	Canada
SAMN13556354	1970	NA [b]	A	Municipal wastewater	2016	Czech Republic
SAMEA1324851	1421	ST46 Cplx	A	NS	2012	NA[c]
SAMEA1324881	174	NA [b]	G	NS	2012	NA[c]
SAMEA1324840	117	NA [b]	G	NS	2012	NA[c]

[a], data provided by the present study, [b] Not applicable, [c] data not available

Table S3: Circularized *bla*$_{CTX-M-1}$-encoding plasmids IncI1-ST3 included in this study.

Accession number	ID	Sample source	Year	Country
SAMN14226788	pH2291-112	Human	2013	Switzerland
SAMN14226492	pHV295	Human	2013-2014	Switzerland
SAMN14227914	pESBL20150097	Human	2015	Denmark
SAMN14227913	pESBL20150178	Human	2015	Denmark
SAMEA6471236 [a]	pJ31	Wild animal (Wild boar)	2006	Portugal
SAMEA6471229 [a]	p2-101	Wild animal (Bird of prey)	2008	Portugal
SAMN14226780	pC59-112	Food producing animal (chicken)	2012	Switzerland
SAMN14226782	pC60-108	Food producing animal (chicken)	2013	Switzerland
SAMN14226779	pC49-108	Food producing animal (chicken)	2013	Switzerland
SAMN14224470	pESBL-305	Food producing animal (chicken)	2014	Netherlands
SAMN14226373	p369	Food producing animal (chicken)	2014	France
MN419433	p20426	Food producing animal (chicken)	2016	Norway
MN419434	p21249	Food producing animal (chicken)	2016	Norway
MN419436	p22440	Food producing animal (chicken)	2016	Norway
MN419437	p22638	Food producing animal (chicken)	2016	Norway
MN419438	p24003	Food producing animal (chicken)	2016	Norway
SAMN14226494	pHV114	Food producing animal (broiler)	2013-2014	Switzerland
SAMN14226493	pHV292	Food producing animal (broiler)	2013-2014	Switzerland
SAMN07197440	pCOV31	Food producing animal (gallus)	2010-2012	France

SAMN07197441	pCOV32	Food producing animal (gallus)	2016	France
SAMN09867582	p04-021	Food producing animal (pig)	2004	France
SAMN03067748	FAP1 plasmid 2	Food producing animal (pig)	2011	Netherlands
SAMN14227733	pCTXM1-MU2	Food producing animal (pig)	2014	Australia
SAMN14227922	p199	Food producing animal (pig)	2015	Denmark
SAMN14227919	p15090172	Food producing animal (pig)	2015	Denmark
SAMN09867573	p17-348F	Food producing animal (pig)	2017	France
SAMN09867593	p17-461F	Food producing animal (pig)	2017	France
SAMN02390175	pTC_N40607	Food (chicken)	2012	USA
SAMN14227924	p14019095	Food (chicken)	2014	Denmark
SAMN14227921	p14006165	Food (chicken)	2014	Denmark
SAMN14227920	p14011252	Food (chicken)	2014	Denmark
SAMN14227918	p15095941	Food (chicken)	2015	Denmark
SAMN14227917	p15124679	Food (duck)	2015	Denmark
SAMN14227923	p15076331	Food (pork)	2015	Denmark
SAMN14228301	p2305	Domestic animal (dog)	2012-2016	Switzerland
SAMEA104588824	RCS34_p	NA [c]	NA [c]	France
SAMEA104588796	RCS72_pI	NA [c]	NA [c]	France
SAMEA104588798	RCS75_pI	NA [c]	NA [c]	France
SAMEA104588797	RCS76_pI	NA [c]	NA [c]	France

[a], this study ; [c], data not available.

Figure S1: Frequency (a) and correlation index (b) of antibiotic resistance genes in non-redundant *E. coli* harboring *bla*$_{\text{CTX-M-1}}$ and IncI1-ST3 plasmids.

a

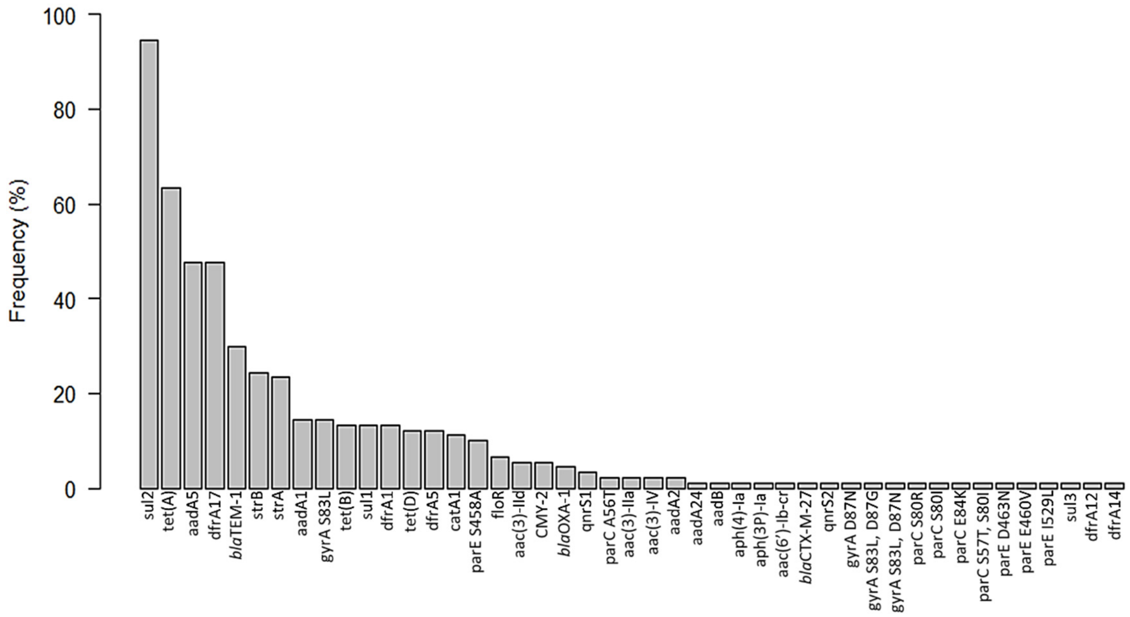

b

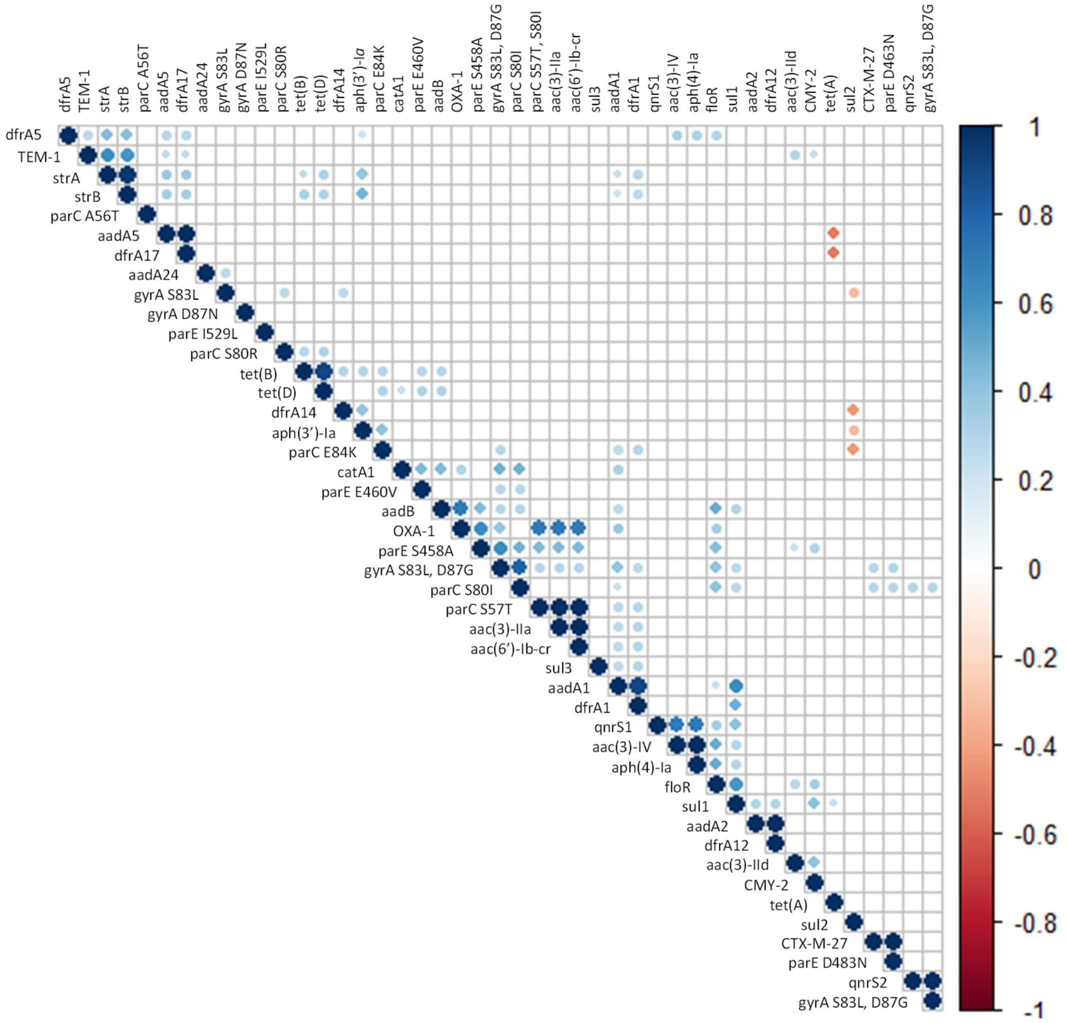

Figure S2: Multiple corresponding analysis performed from the synteny of shufflon region of *bla*$_{CTX-M-1}$ IncI1-ST3 plasmids, which were extracted from 77 WGSs. The resulting clusters are surrounded by 95% confidence ellipses and are supported by stastistic tests (Adonis test's p-value: <0.001 and R2: 0.98). The most contributory genetic features are indicated in red (B#: shuffling segment B ; #: position of the genetic feature, rv: reverse and fd: forward).

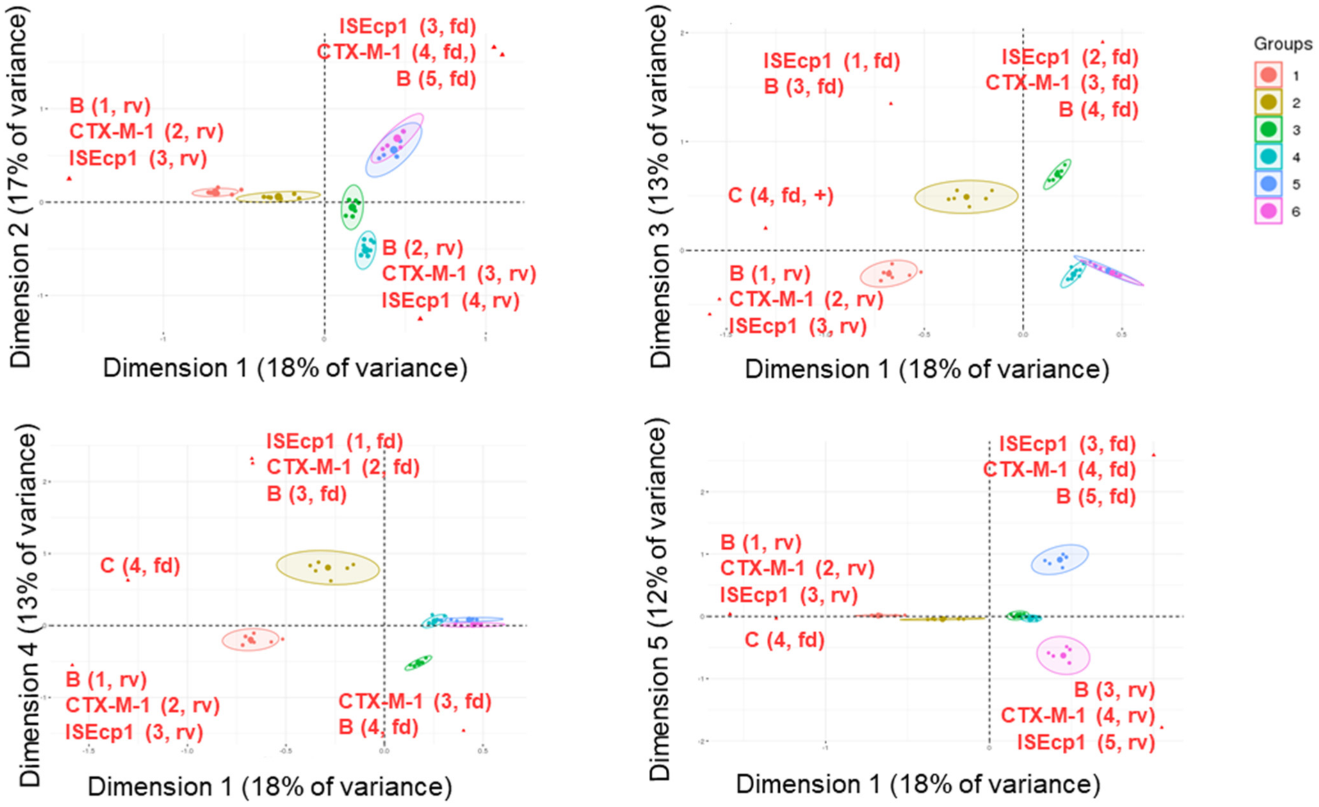

Figure S3: Heatmap showing the presence (red) and the absence (blue) of synteny blocks in the shufflon region of plasmids IncI1-ST3-$bla_{\text{CTX-M-1}}$. The dataset was extracted from WGSs containing assembled shufflons (referred to as biosample accession number). The genetic features of shufflons are indicated (A: shufflon segment A, B: shufflon segment B, C: shufflon segment C, CTX-M-1: $bla_{\text{CTX-M-1}}$ and IS*Ecp1*) with the position of the genetic feature in the synteny (index number) and its orientation (rv: reverse and fd: forward) as well as metadata and the clusters derived from MCA and HC analysis (C#).

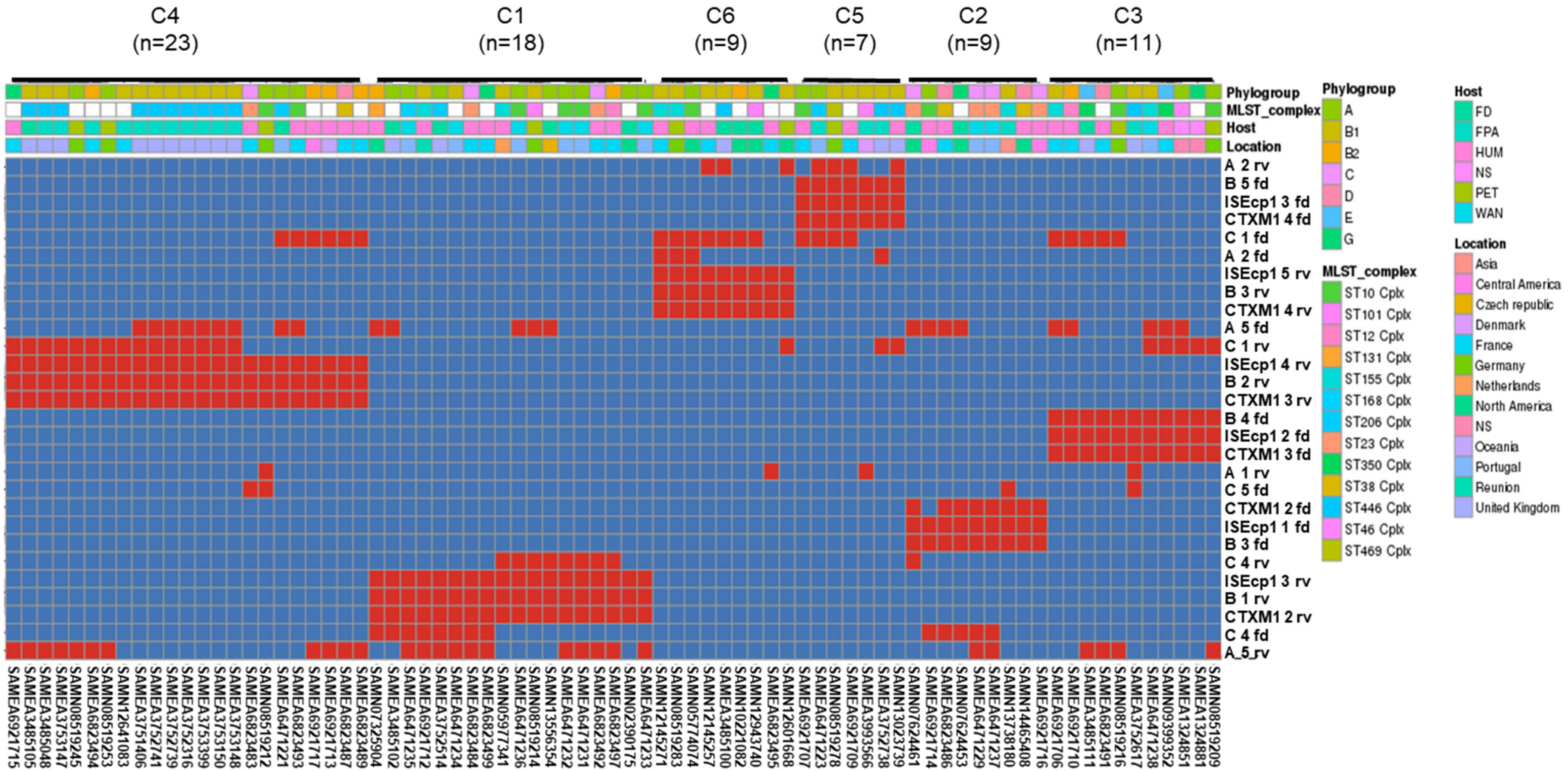

Figure S4: Hierarchical classification of plasmids IncI1-ST3-*bla*$_{CTX-M-1}$ based on the synteny of the shufflon region. The dataset was extracted from WGSs containing assembled shufflons (referred to as biosample accession number). The synteny is reported with the genetic features indicated as arrows (orange: recombinase gene *rci*, mauve: shufflon segment, blue: shufflon segment B, black: IS*Ecp1*, red: *bla*$_{CTX-M-1}$, green: shufflon segment, violet: *pilV* and yellow: *pilRST*). The shufflons having identical synteny are surrounded by black boxes and those with identical location and source are highlighted. The table reports geographic origin (AS: Asia, CA: Central America, CZ: Czech Republic, DE: Germany, FR: France, GU: Guyana, NA: North America, NC: New Caledonia, NL: Netherlands, PT: Portugal and UK: United-Kingdom) and the sample source (HUM: human, FD: food, FPA: food-producing animal, PET: companion animal and WAN: wild animal).

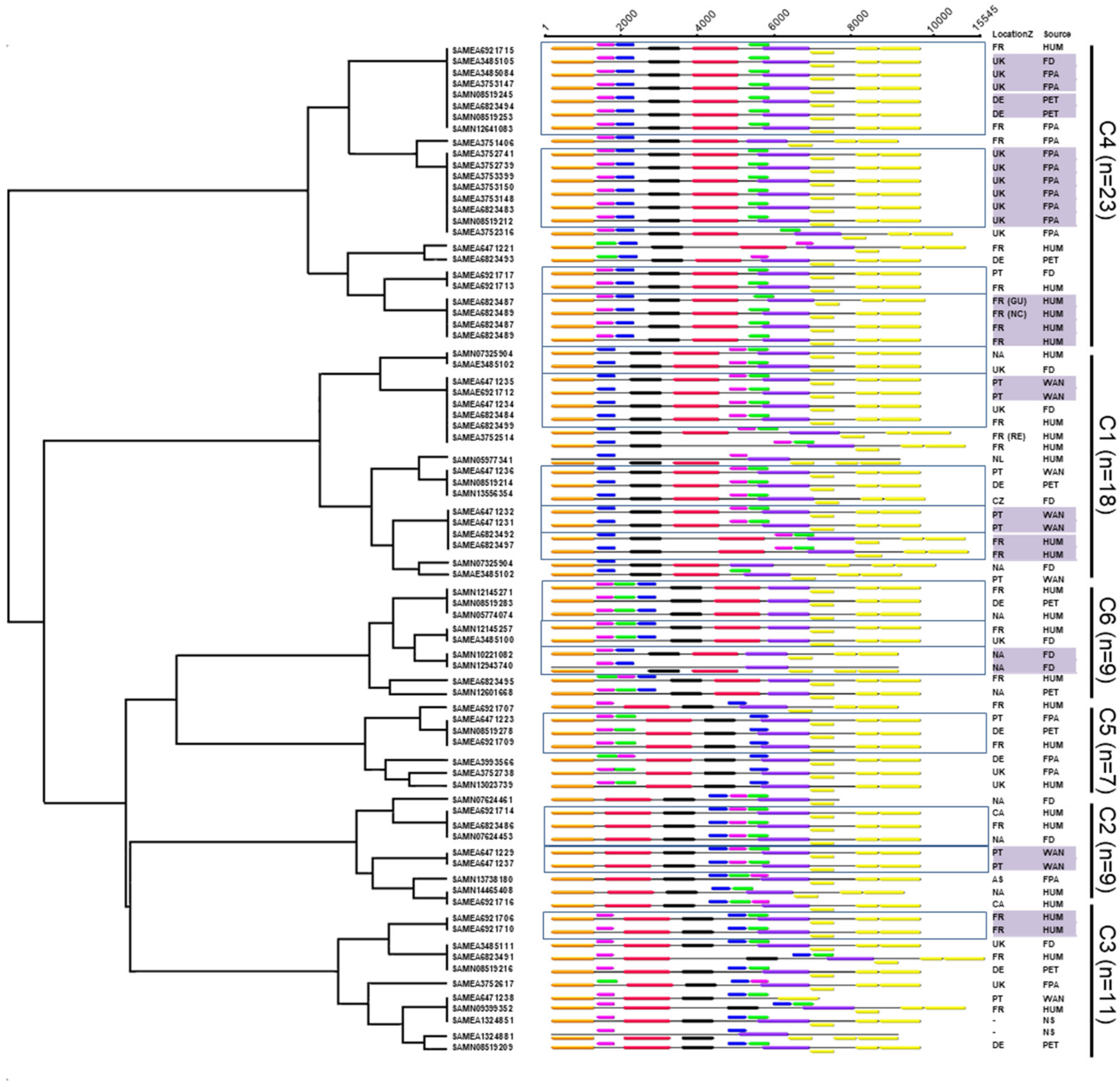

Supplement: Supplementary file 1 [file microorganisms-09-01471-s001.zip › microorganisms-1289184-supplementary.pdf]
